# *Burkholderia cepacia* meningitis in the Central African Republic

**DOI:** 10.11604/pamj.2019.32.12.16552

**Published:** 2019-01-07

**Authors:** Thierry Frank, Alain Farra, Pierre-Alain Rubbo, Jean-Robert Mbecko, Hugues Sanke, Anne Le Flèche-Matéos, Jean-Pierre Lombart, Alain Berlioz-Arthaud

**Affiliations:** 1 Unit of Bacteriology, Pasteur Institute of Bangui, Bangui, Central African Republic; 2 Environment and Infectious Risk Unit, Laboratory for Urgent Response to Biological Threats, Pasteur Institute, Paris, France

**Keywords:** Meningitis, Burkholderia cepacia, antibiotics, Central African Republic

## Abstract

Burkholderia cepacia causes frequent infections in immunocompromised and hospitalized patients, with a significant mortality rate. This bacterial species has also been associated with epidemic outbreaks due to contamination of antiseptic solutions and parenteral and nebulized medications. In 2016, in the town of Bongonon in the north of the Central African Republic (CAR), a three-year-old boy with febrile meningeal syndrome (fever, neck stiffness and altered general condition) was admitted for a medical consultation provided by the nongovernmental organization MSF-Spain. On 20 March 2016, a sample of the boy’s cerebrospinal fluid was sent to the Bacteriology Laboratory of the Pasteur Institute of Bangui for analysis. Conventional bacteriology showed that the isolate was a Gram-negative bacillus, which was identified as B. cepacia by using API 20 NE, with 99.9%confidence. In addition, the strain presented an acquired resistance to ticarcillin-clavulanate, ceftazidime and imipenem but remained susceptible to cotrimoxazole. As B. cepacia had never previously been isolated from cerebrospinal fluid in Africa, we chose to identify the strain by 16S rRNA gene sequencing. The molecular data showed that the isolate belonged to B. cepacia group. This is the first report of a case of meningitis caused by B. cepacia in CAR and developing countries.

## To the editors of the Pan African Medical Journal

Bacteria in the *Burkholderia* genus are ubiquitous Gram-negative organisms that can cause a number of diseases in animals and humans [1]. *Burkholderia cepacia* is a serious opportunistic pathogen in immunocompromised and cystic fibrosis patients and has also been linked to healthcare-associated outbreaks due to contamination of antiseptic solutions and parenteral and nebulized medications [2-5]. For many years, *Burkholderia* were recognized as non-fluorescent pseudomonads [1]. Polyphasic taxonomy analyses, including 16S rRNA sequencing, DNA-DNA hybridization and fatty acid analysis, showed that the *Burkholderia* genus comprises seven species in the *Pseudomonas* rRNA group II (*P. cepacia, P. caryophylli, P. gladioli, P. mallei allei, P. pseudomallei, P. solanacearum and P. pickettii*) [1]. Further taxonomic studies allowed designation of binomial species names for clinically isolated *Burkholderia*, which are now referred to as species of the *B. cepacia* complex, composed of at least 17 species, including *B. cepacia, B. multivorans, B. cenocepacia, B. vietnamiensis, B. ambifaria, B. stabilis, B. dolosa, B. anthina and B. pyrrocinia* [1]. In the early 1980s, strains of *B. cepacia* were increasingly being recovered from cultures of respiratory tract specimens from cystic fibrosis patients [1]. Here, we describe the first case of meningitis caused by *B. cepacia* in the Central African Republic.

In 2016, in the town of Bongonon in northern Central African Republic (CAR), a 3-year-old boy with febrile meningeal syndrome (fever, neck stiffness and altered general condition) was admitted for a medical consultation organized by the non-governmental organization MSF-Spain. On 20 March 2016, a sample of the boy's cerebrospinal fluid (CSF) was sent to the Bacteriology Laboratory of the Pasteur Institute of Bangui for analysis. The sample was analysed by conventional bacteriology, including direct examination, Gram staining, culture, oxidase test and API 20 NE strips (BioMérieux, Marcy l'Étoile, France). Antibiotic susceptibility was determined by the disc diffusion method (Bio-Rad, Marnes-la-Coquette, France) on Mueller-Hinton agar and interpreted according to the recommendations of the “Comité de l'Antibiogramme de la Société Française de Microbiologie”. CSF protein and glucose were determined by ABX PENTRA C400- HORIBA. As the blood sample has not been taken, we could not determine the HIV status of the patient.

**Figure 1:**
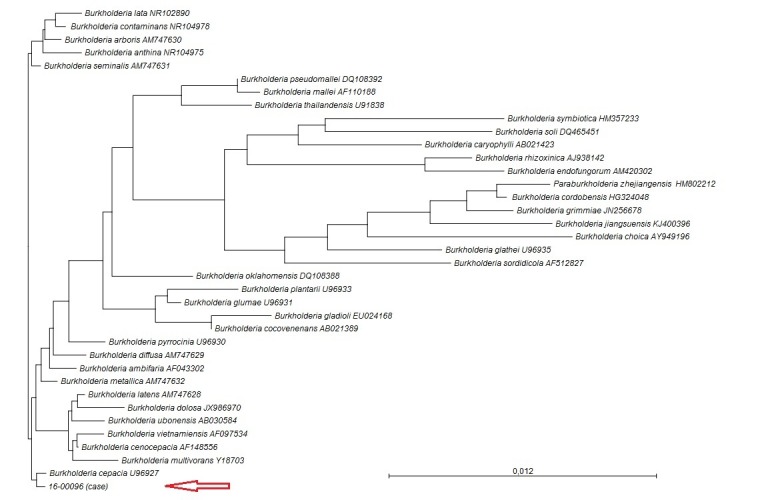
Phylogenetic tree based on 16S rRNA gene sequences of isolate 16_00096 and representative Burkholderia species, contructed with the neighbour-joining method; Bootstrap values > 50% (based on 1000 replicates) are given at branching points. All sequences are from type strains

The sample was slightly turbid and consisted of numerous leukocytes (225/mm^3^) and red cells (144/mm^3^).The leukocytes comprised neutrophils (50%) and lymphocytes (50%). The patient had high proteinuria (0.84g/L) and extremely low glycorachia (0.01 mmol/L). The cytological and biochemical data were consistent with bacterial meningitis. The isolate was a Gram-negative bacillus identified as *Burkholderia cepacia* by API 20NE with 99.9% of confidence (Table 1). The strain was resistant to piperacillin, ticarcillin-clavulanate, ceftazidime, cefepime, imipenem but remained susceptible to cotrimoxazole. As *B. cepacia* has never been isolated from CSF in Africa, we chose to identify the strain by molecular typing of DNA extracted from colonies of the CSF. Our isolate (labelled11_00096) was cultured on tryptocasein soy agar at 30°C. After 24h, circular, yellow colonies were seen. The complete sequence of the rrs gene (encoding 16S rRNA) was determined by the classic Sanger method, as described elsewhere and consisted of 1459bp of continuous nucleotides [6]. The sequence was compared with all the bacterial sequences in the GenBank database with the BLAST program. A phylogenetic tree was constructed with CLC Main Workbench software, version 7.6.4 (Qiagen, Germantown, MD, USA) with the neighbour-joining algorithm, which showed that the isolate belonged to the *Burkholderia cepacia* group (Figure 1). Apart from respiratory tract infections, a case of multiple liver abscesses due to *B. Cepacia* has been reported [7]. We report the first case of meningitis caused by *B. cepacia* in developing countries. This paper describes a case of meningitis due to multidrug-resistant *B. Cepacia* in CAR. The emerging isolate was resistant to most of the antibiotics except cotrimoxazole. Further studies are warranted to understand the reasons for this emergence.

**Table 1:** Biochemical characteristics of isolate 16_00096 according to API 20 NE

Reaction and enzymes	Results	Substrate	Assimilation
Reduction of nitrates to nitrites	+	D-Glucose	+
Indole production	-	L-Arabinose	+
D-Glucose fermentation	-	D-Mannose	+
Arginine dihydrolase	-	D-Mannitol	+
Urease	-	*N*-Acetylglucosamine	+
β-Glucosidase	+	D-Maltose 1.4	-
Protease	+	Potassium gluconate	+
β-Galactosidase	+	Capric acid	+
Cytochrome oxidase	+	Adipic acid	+
-	-	Malic acid	+
-	-	Trisodium citrate	+
-	-	Phenylacetic acid	+

**Ethical approval:** we confirm that ethical approval for any human or animal experimentation was obtained from an accredited ethical committee.

## Competing interests

The authors declare no competing interests.

## References

[1] Choudhary KS, Hudaiberdiev S, Gelencser Z, Goncalves Coutinho B, Venturi V, Pongor S (2013). The organization of the quorum sensing luxI/R family genes in Burkholderia. Int J Mol Sci.

[2] Zurita J, Mejia L, Zapata S, Trueba G, Vargas AC, Aguirre S (2014). Healthcare-associated respiratory tract infection and colonization in an intensive care unit caused by Burkholderia cepacia isolated in mouthwash. Int J Infect Dis.

[3] Hamill RJ, Houston ED, Georghiou PR, Wright CE, Koza MA, Cadle RM (1995). An outbreak of Burkholderia (formerly Pseudomonas) cepacia respiratory tract colonization and infection associated with nebulized albuterol therapy. Ann Intern Med.

[4] Doit C, Loukil C, Simon AM, Ferroni A, Fontan JE, Bonacorsi S (2004). Outbreak of Burkholderia cepacia bacteremia in a pediatric hospital due to contamination of lipid emulsion stoppers. J Clin Microbiol.

[5] Nasser RM, Rahi AC, Haddad MF, Daoud Z, Irani-Hakime N, Almawi WY (2004). Outbreak of Burkholderia cepacia bacteremia traced to contaminated hospital water used for dilution of an alcohol skin antiseptic. Infect Control Hosp Epidemiol.

[6] Le Fleche-Mateos A, Levast M, Lomprez F, Arnoux Y, Andonian C, Perraud M (2015). Rouxiella chamberiensis gen nov, sp nov, a member of the family Enterobacteriaceae isolated from parenteral nutrition bags. Int J Syst Evol Microbiol.

[7] Mukhopadhyay C, Bhargava A, Ayyagari A (2004). Two novel clinical presentations of Burkholderia cepacia infection. J Clin Microbiol.

